# Lateralized nigrostriatal dopamine pathway activation promotes early reversal learning

**DOI:** 10.3389/fnbeh.2025.1703094

**Published:** 2025-12-10

**Authors:** Stefan W. Fleps, Ben Yang, Nicolette A. Moya, Xunhui Wu, Seongsik Yun, Jones G. Parker

**Affiliations:** Department of Neuroscience, Northwestern University, Chicago, IL, United States

**Keywords:** dopamine, reversal learning, dorsal striatum (DS), substantia nigra, optogenetics

## Abstract

The dorsomedial striatum (DMS) is believed to promote action-outcome associations by integrating cortical and limbic afferents with nigrostriatal dopamine. This functional role for the DMS encompasses reinforcement learning processes traditionally ascribed to the ventral (outcome valuation) and lateral (action-selection) subdivisions of the striatum. Previous studies have shown that DMS dopamine signaling encodes actions and outcomes, often in a lateralized manner (e.g., dopamine release is greater for contralateral actions). To determine how these dynamics evolve with changing action-outcome contingencies, we recorded dopamine axon Ca^2+^ activity in the DMS during a lateralized reversal learning task in mice. Using a miniaturized fluorescence microscope, we found that dopamine axon Ca^2+^ activation in the DMS encoded actions and outcomes with a lateral bias, but only very early in reversal learning, immediately after the action-outcome contingency switch. Specifically, we found that dopamine axon activation during contralateral choices and the rewards associated with those choices were only greater than ipsilateral choices and their rewards in the first session of reversal. Over the course of reversal, reward- and choice-evoked dopamine axon activation subsided for contralateral, but not ipsilateral, choices, resulting in no lateral bias after learning the new action-outcome contingency. Consistent with a causal role for these lateralized dynamics, unilateral optogenetic inhibition of the nigrostriatal dopamine pathway impaired contralateral reversal by reducing “win-stay” responses. However, this deficit was transient, occurring only during the first reversal session. Our results suggest that dopamine signaling in the DMS facilitates the exploration of new actions, specifically at the specific moment the previous action-outcome contingency becomes conflicted. In our lateralized reversal learning task, this facilitation was particularly important when the new action and resulting outcome were contralateral to the recorded hemisphere. These findings advance our understanding of how the DMS carries out its ascribed role in action-outcome learning.

## Introduction

Dopamine signaling in different sub-regions of the striatum is believed to subserve distinct aspects of reinforcement learning (Belin and Everitt, 2008; Burton et al., 2015; Everitt and Robbins, 2016). For instance, ventral striatum dopamine signaling encodes the valence, magnitude, and predictability of reinforcers, which are believed to be important for forming “stimulus-outcome” associations (e.g., Pavlovian conditioning). By contrast, dorsolateral striatum dopamine signaling correlates with movement and is canonically implicated in the formation and maintenance of “stimulus–response” associations. Interfaced between the ventral and dorsolateral striatum, the dorsomedial striatum (DMS) receives both limbic and motor inputs, positioning the DMS to facilitate the formation of “response-outcome” associations.

Reversal learning has provided many insights about the neural substrates of action selection and the dopamine system’s role in this process (Izquierdo and Jentsch, 2012; Izquierdo et al., 2017). The DMS is particularly implicated in reversal learning. Inactivating or lesioning the DMS impairs reversal learning in rodents and primates (Ragozzino and Choi, 2004; Ragozzino, 2007; Clarke et al., 2008; Castañé et al., 2010). Dopaminergic denervation of the DMS or manipulations of its dopamine receptor-expressing neurons also impair reversal learning (Grospe et al., 2018; Peak et al., 2020).

To better understand how DMS dopamine may encode changing action-outcome contingencies, we used a miniature microscope to record dopamine axon Ca^2+^ dynamics during a T-maze reversal learning task. We focused our analysis on these dynamics during the action (i.e., choice) and outcome over the course of reversals to the right and then the left arms of the maze. Similar to previous related studies, dopamine axon activation was greater for contralateral than for ipsilateral choices. However, this effect was observed only immediately following reversal to the maze arm contralateral to the imaged striatal hemisphere. Once the new action-outcome contingency was acquired, contralateral choice-evoked dopamine axon activation subsided to become indistinguishable from ipsilateral choice-evoked responses. This choice-related lateralization of dopamine signaling was absent for forced trials, suggesting dependence on choice volition. We also found that reward-related dopamine axon activation was greater following contralateral choices in early reversal learning, though this was also true on forced trials. This context-specific, contralateral bias in dopamine axon transmission may facilitate new actions, specifically at the time the previous action-outcome contingency becomes conflicted. Consistent with this interpretation, we found that unilaterally inhibiting dopamine impaired early, but not later, reversal learning when contralateral choices became newly rewarded. Our results shed new light on how the DMS influences action-outcome learning to facilitate behavioral flexibility.

## Materials and methods

### Animals

We housed and handled all mice according to guidelines approved by the Northwestern University Animal Care and Use Committee. Animal housing rooms were maintained at 70–74 °F and 30–70% humidity. We used both male and female mice housed on a 12-h light/dark reverse light cycle. We performed all experiments during the dark phase. We used heterozygous *Slc6a3*^IRES-Cre^ mice (Bar Harbor, ME #0006660) to express GCaMP or ArchT in SNc dopamine neurons. Although we did not utilize these Cre-drivers in these experiments, the mice used for *in vivo* imaging (but not optogenetics) were also heterozygous for either *Adora2a*^Cre^ (KG139) or *Drd1*^Cre^ (FK150) BAC transgenes from the Mutant Mouse Regional Resource Centers (MMRC). All mice were maintained on a C57BL/6 J background (Jackson Labs #000664) and were at least 8 weeks of age at the beginning and < 36 weeks of age at the end of their experiment. We euthanized all experimental mice using isoflurane with the bell jar method followed by thoracotomy and perfusion. We euthanized all other mice using CO_2_ at a flow rate calculated to displace 30–70% of the air volume in the chamber per minute, followed by cervical dislocation.

### Virus injection surgeries

We anesthetized mice with isoflurane (2% in O_2_) and stereotaxically injected virus at a rate of 250 nL·min^−1^ into the SNc using a microsyringe with a 33-gauge beveled-tip needle (WPI, Nanofil). We injected 500 nL of each virus (all from Watertown, MA) at the following final dilution concentrations (GC·mL^−1^): AAV2/5-hSyn-FLEX-axon-GCaMP6s: 5.4 × 10^12^, AAV2/1-hSyn-FLEX-jRGECO1a: 2.6 × 10^13^; AAV2/5-hSyn-FLEX-mCherry: 1.7 × 10^12^; AAV2/5-CAG-FLEX-ArchT-tdTomato: 1.1 × 10^12^. All anterior–posterior (AP), medial-lateral (ML), and dorsal-ventral (DV) coordinates are reported in mm from bregma unless otherwise noted. All injections and implants were in the left hemisphere. For all DV coordinates, we went 0.5-mm past the injection target and then withdrew the syringe back to the target for the injection. After each injection, we left the syringe in place for 5 min, withdrew the syringe 0.1 mm, waited 5 min more, and then slowly withdrew the syringe. For the optogenetic experiments, we subsequently implanted the fiber optic during the same surgery (see below). For Ca^2+^ imaging experiments, we sutured the scalp, injected analgesic (Buprenorphine SR, 1 mg·kg^−1^), and allowed the mice to recover for at least 1 week before optical implant surgery. For the Ca^2+^ imaging experiments, we injected AAV2/5-hSyn-FLEX-axon-GCaMP6s unilaterally in the SNc (AP: (bregma-to-lambda distance in mm)/4.21 × 3.5, ML: −1.3 mm, DV: −4.0 mm) and AAV2/1-hSyn-FLEX-jRGECO1a in the DMS (AP: 0.5; ML: -1.5; DV: −2.7). Note that the DMS expression of jRGECO1a was for separate experiments and was not imaged or analyzed in the current study. For animals used for optogenetic inhibition experiments, we unilaterally injected AAV2/5-hSyn-FLEX-mCherry (control), or AAV2/5-CAG-FLEX-ArchT-tdTomato at the same SNc coordinates.

### Optical implant surgeries

For the Ca^2+^ imaging experiments, we constructed optical guide tubes by using ultraviolet (UV) liquid adhesive (Jamesburg, NJ #81) and a UV spot curing system (Longview, TX) to fix a 2-mm-diameter disc of #0 glass (Phoenix, AZ) to the tip of a 3.8-mm-long, 18-gauge, extra-thin stainless-steel tube (Sparta, TN). We ground off any excess glass using a polishing wheel (Santa Ana, CA).

To prepare mice for Ca^2+^ imaging, we anesthetized virus-injected mice with isoflurane (2% in O_2_) and used a 1.4-mm-diameter drill bit to create a craniotomy (AP: 0.5; ML: −1.5) for implanting the optical guide tube. We used a 0.5-mm-diameter drill bit to drill four additional small holes at spatially distributed locations for insertion of four anchoring skull screws (#AMS120/2–000-120 × 5/64 SL FILLISTER; Fallbrook, CA). We aspirated the cortex down to DV: −2.1 mm from dura using a 27-gauge blunt-end needle and implanted the optical guide tube at DV: −2.35 mm from dura. After placing the guide tube, we applied Metabond (Parkell, Edgewood, NY) to the skull and then used dental acrylic (Cuyahoga Falls, OH) to fix the full assembly, along with a custom stainless steel headplate (Milpitas, CA) for head-fixing mice during attachment and release of the miniature microscope. We injected analgesic (Buprenorphine SR, 1 mg·kg^−1^) and allowed the mice to recover for 3–4 weeks before imaging fluorescence in the striatum and subsequently mounting the miniature microscope.

To prepare mice for optogenetic manipulation, in anesthetized mice, immediately after virus injection, we also applied skull screws as described above and lowered a fiberoptic cannula (diameter: 200 μm; length: 6.5 mm; NA: 0.66; Holon, Israel) through the virus-injection craniotomy to DV: −3.9 mm. We then applied Metabond, a headplate, dental acrylic, and administered analgesic and allowed the mice to recover as described above.

### Miniature microscope mounting

We head-fixed each implanted mouse by its headplate on a running wheel and inserted a gradient refractive index (GRIN) lens (1-mm diameter, ~4.12-mm length, 0.46 numerical aperture (NA), 0.45 pitch; Mountain View, CA) into the optical guide tube. We then assessed axon-GCaMP6s expression in the DMS using a commercial two-photon fluorescence microscope (Madison, WI). Subsequently, we anesthetized mice with ample GCaMP6s expression (2% isoflurane in O_2_), placed them into a stereotaxic frame, and glued the GRIN lens in the guide tube with UV-curable epoxy (Loctite, no. 4305, Henkel, Elgin, IL). Next, we used the stereotaxic manipulator to lower the miniature microscope with its attached base plate (nVista, Inscopix) toward the GRIN lens until the fluorescent tissue came into focus. We then created a structure of blue-light curable resin (Flow-It ALC, Orange, CA) on the dental acrylic skull cap around the base plate and attached the structure to the miniature microscope base plate using UV curable epoxy. Finally, we coated the epoxy/resin with black nail polish to make it opaque.

### *In vivo* Ca^2+^ imaging in an open field

We habituated mice to a circular open field arena (30.48 cm-diameter) for 3 days for 1 h on each of the 3 days. Right before each imaging session, we head-fixed each mouse by their headplate on a running wheel to attach the miniature microscope. We adjusted the focal plane using Inscopix Data Acquisition Software and then released the mouse and placed them into the open field arena. After 20 min of habituation, we recorded Ca^2+^ activity for 1 h in the open field. We used an illumination power of 50–200 μW at the specimen plane and a 20-Hz image frame acquisition rate.

### Behavioral tracking in an open field

We used a Transistor-Transistor Logic-triggered video camera with IC Capture software (Charlotte, NC) and a varifocal lens (T3Z2910CS, NY, NY) to record 20-Hz videos of mouse behavior synchronized to neural recordings. We used software written in ImageJ to track each mouse’s position in the open field arena. In brief, we used this software to identify the mean location of the largest and darkest contiguous pixel group (that is, the mouse) in each movie frame and then computed the mouse’s locomotor speed from the trajectory of its centroid location across movie frames. We processed the resulting data using custom Matlab code and applied a 1-s median filter to the resulting speed trace.

### Ca^2+^ movie processing

For the 20-Hz miniature microscope recordings in both the open field and T-maze, we used the CIAtah analysis suite^1^ (Corder et al., 2019) to spatially downsample the raw movies 4x and motion correct using the TurboReg algorithm (Thevenaz et al., 1998). We then used custom Matlab code to (1) correct the movies for any photobleaching using an exponential fit to the time trace of the average of every pixel in every frame. We then computed the mean value of all the averaged frames, subtracted that value from the trace of each frame’s average, and used an exponential to fit the ‘baseline-corrected’ trace (using the ‘fit’ function in Matlab with ‘exp2’). We then subtracted the mean of all pixels across frames from each pixel in each frame and divided each pixel in each frame by the computed fit. We then (2) computed the Δ*F/F* of the corrected movie by subtracting each pixels average over the entire recording session and dividing by that average in each frame, (3) used a manually drawn rectangular mask to select the center area of the GRIN lens and exclude areas around the edges of the lens, and then (4) averaged all of the pixels contained within that mask to calculate the overall mean Δ*F/F* trace.

### *In vivo* Ca^2+^ imaging during reversal learning

We individually housed and gradually food-restricted mice to 85% of their *ad libitum* body weight. We then habituated the mice to an automated T-maze (Skokie, IL) for 2 days. During the habituation days, we placed mice in the maze and allowed them to freely explore and forage for 45 mg chocolate-flavored pellets (Bio-Serv) sporadically placed throughout the maze for 30 min. On the subsequent 2 days, we pre-trained mice in sessions consisting of 10 alternating (left vs. right) forced trials from the center holding area to trigger reward pellet delivery in the goal arm and return to the center holding area. The day after pre-training, we began training mice in a reversal learning task using both forced and choice trials in each direction. In particular, mice received 4 training blocks consisting of 5 choice trials followed by 2 forced trials (one in each direction). During this phase, entry into only one of the arms resulted in pellet delivery (28 total trials). Once mice made ≥75% correct choices on two consecutive days, they had reached criterion, and we reversed which arm was rewarded on the subsequent day. On the first day of reversal, mice underwent two blocks (5 choices then two forced in each direction) of training (14 total trials) to the side that they had previously reached criterion on, followed by 4 blocks of training where the rewarded arm was reversed to the opposite arm on which they were initially trained. On subsequent days, mice underwent 4 blocks of training until they reached criterion on the newly rewarded side (≥75% correct of the choice trials). For every mouse, we initially rewarded the arm that was ipsilateral to the imaged hemisphere (the left arm), and the reward arm was reversed to the arm contralateral to the imaged hemisphere (the right arm) after the mice reached criterion during initial acquisition.

After the second day of habituation, for each session (pre-training, initial acquisition, and the two reversals), we fixed the miniature microscope to the head of the mouse by head-fixing it by their headplate to a running wheel. After attaching the miniature microscope, we adjusted its focal plane using Inscopix Data Acquisition Software and released the mouse after securing the microscope. We recorded and processed movies of Ca^2+^ throughout initial acquisition and reversal using the same procedure described for open field recordings. To synchronize the neural recordings, we used a logic analyzer (Leander, TX) to read the miniature microscope frame clock and TTL pulses triggered by maze events and infrared beam breaks in the maze. We used custom Matlab scripts to process these synchronized outputs and time-lock the Δ*F/F* trace to task epochs (e.g., choice turn start and completion). We computed the choice duration as the median time for each mouse between turn start and completion for the different choice and forced choice trial categories. We used the median (as opposed to mean) to exclude spurious outlier values and then averaged the median values for each trial type across mice.

### Optogenetic inhibition during reversal learning

We followed the same habituation and pre-training protocol as for *in vivo* imaging. Then, mice underwent their initial acquisition training sessions consisting of 25 choice trials in which the left arm (ipsilateral to the implanted hemisphere) of the T-maze was rewarded. Once mice made ≥75% correct choices on at least 2 consecutive days, they were then considered to have reached a criterion, and we reversed which arm was rewarded on the subsequent day. On the first day of reversal to the right arm, the mice underwent a training block of 10 choice trials in which the left arm (prior association) was rewarded, followed by 25 choice trials in which the right arm (contralateral to the implanted hemisphere) was rewarded. We continued training mice with the right arm rewarded for 25 choice trials per session until they reached the criterion.

Before each session, we fixed an optical fiber patch cord (Quebec City, Quebec) to the implanted fiberoptic ferrule, while mice were briefly head fixed on a running wheel. We then released the mice and placed them in the maze. Although we did attach the patch cable during habituation, pre-training, or initial acquisition, we did not apply light-emitting diode (LED) stimulation until the reversal. Beginning on the first day of reversal, we used the Prizmatix Dual-Optogenetics-LED to provide high-power light to activate ArchT expressed in SNc dopamine neurons (545 nm wavelength, constant LED, with a power of 10 mW at the implanted fiberoptic ferrule tip). We confirmed the power settings each day before training the mice. We used an Monza, Italy to trigger LED stimulation during the behavioral sessions. In particular, LED stimulation started when mice broke the first infrared beam in the maze (corresponding to the choice turn start) directly following the release from the center holding area. LED stimulation lasted for 5 s from the start of the turn for every choice and then for another 5 s upon turn completion, only if the mouse correctly chose the right (contralateral) arm.

We computed the “Win-Stay” choices as the percentage of correct choices following a correct choice and the “Lose-Shift” choices as the percentage of correct choices following an incorrect choice in 5-trial bins. We computed perseverative errors as ≥ 3 incorrect choices in a 5-trial block until a 5-trial block with < 3 incorrect choices. We computed regressive errors as any incorrect choice after a 5-trial block with < 3 incorrect choices.

### Histology

After all T-maze experiments, we euthanized and intracardially perfused mice with PBS followed by a 4% solution of paraformaldehyde in PBS. After perfusion, we extracted the brains and placed them in 4% paraformaldehyde for 1–3 days. We sliced 50–70-μm thick coronal brain sections using a vibratome (Leica VT1000s). For the mice used for *in vivo* imaging, we immunostained for axon-GCaMP6s in DMS and SNc using an anti-GFP antibody (1:1000, Invitrogen, A11122) and a fluorophore-conjugated secondary antibody (1:500, Jackson Immunoresearch 711–546-152). For the mice used for *in vivo* imaging, we immunostained for tyrosine hydroxylase (1:500; Aves TYH) and fluorophore-conjugated secondary antibodies (1:500, Jackson Immunoresearch and 711–546-152 or 715–586-150, respectively). We mounted the sections and imaged fluorescence using a slide-scanning fluorescence microscope (Leica Thunder). We reported optical implant placements in mouse brain atlas sections (Franklin and Paxinos, 2001).

## Results

### Endoscopic recordings of dopamine axon Ca^2+^ activity using a miniature microscope

**Figure 1:**
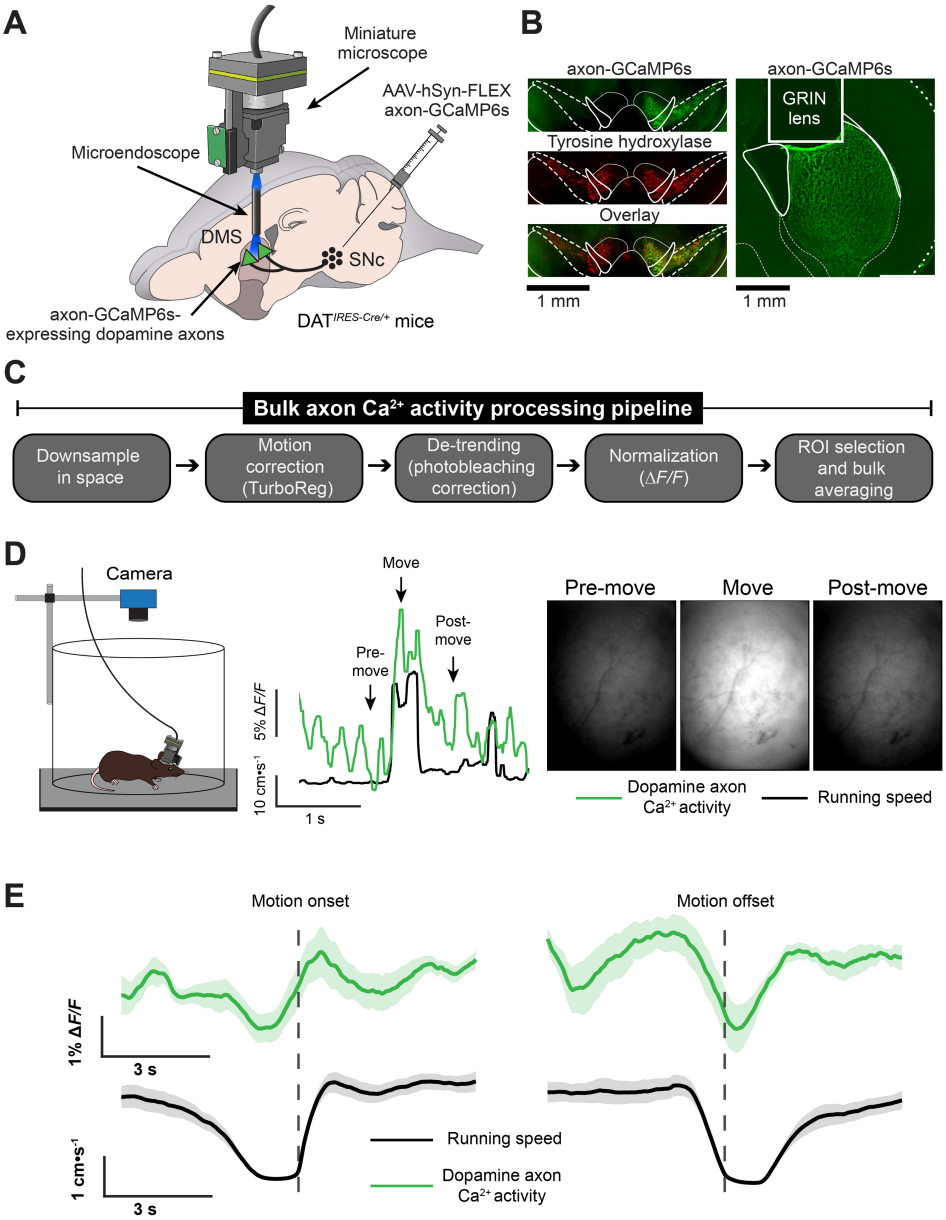
Imaging dopamine axon Ca^2+^ activity in freely behaving mice. **(A)** We virally expressed axon-targeted GCaMP6s in the substantia nigra pars compacta (SNc) of DAT-Cre mice and implanted a microendoscope into the dorsomedial striatum (DMS) to image dopamine axon Ca^2+^ activity using a miniature microscope. **(B)** Histological verification of GCaMP6s-expressing dopamine axon terminals at the DMS recording site and at the site of virus injection in the SNc. **(C)** Movie processing steps for extracting the dopamine axon Ca^2+^ signal. **(D)** Dopamine axon Ca^2+^ activity (bulk ∆*F/F*) and running speed, time-locked to a single instance of movement onset in the open field for a representative mouse. The right still images are raw fluorescence images (i.e., before normalization) at the corresponding timepoints indicated in the middle plot. **(E)** Mean ± s.e.m. Dopamine neuron axon Ca^2+^ activity and running speed, time-locked to movement onset (*left*) and offset (*right*), and averaged across all instances and mice (*N* = 8 mice).

We virally expressed axon-GCaMP6s (Broussard et al., 2018) in substantia nigra dopamine neurons of *Slc6a3*^IRES-Cre^ mice (DAT-Cre), implanted a microendoscope into the DMS, and affixed a miniature microscope to image dopamine axon Ca^2+^ activity during behavior (Figure 1A). We histologically verified that this approach resulted in axon-GCaMP6s expression at the DMS imaging site and in the SNc, where expression overlapped with the dopamine cell marker tyrosine hydroxylase (Figure 1B; Supplementary Figure S1).

In a subset of these mice, we recorded dopamine axon activity and behavior during 1-h sessions in an open field arena. We developed a custom processing pipeline for these imaging data based on our earlier studies using miniature microscopes to record bulk- and cellular-resolution Ca^2+^ dynamics in the DMS (Legaria et al., 2022; Yun et al., 2023; Yun and Parker, 2025) (Figure 1C). For this study, we treated the full field fluorescence as a single bulk trace by pre-processing the movies and averaging the pixels within a selected region-of-interest over the Δ*F/F* movie. Consistent with published imaging studies of the nigrostriatal dopamine pathway (Howe and Dombeck, 2016; da Silva et al., 2018), we observed a correspondence between dopamine axon Ca^2+^ activation and movement in individual mice (Figure 1D). When time-locking the bulk Δ*F/F* traces and averaging across all mice, dopamine axon Ca^2+^ activity correlated with running speed during spontaneous motion onset or offset in the open field (Figure 1E).

### Recording dopamine axon Ca^2+^ activity during reversal learning in a T-maze

**Figure 2:**
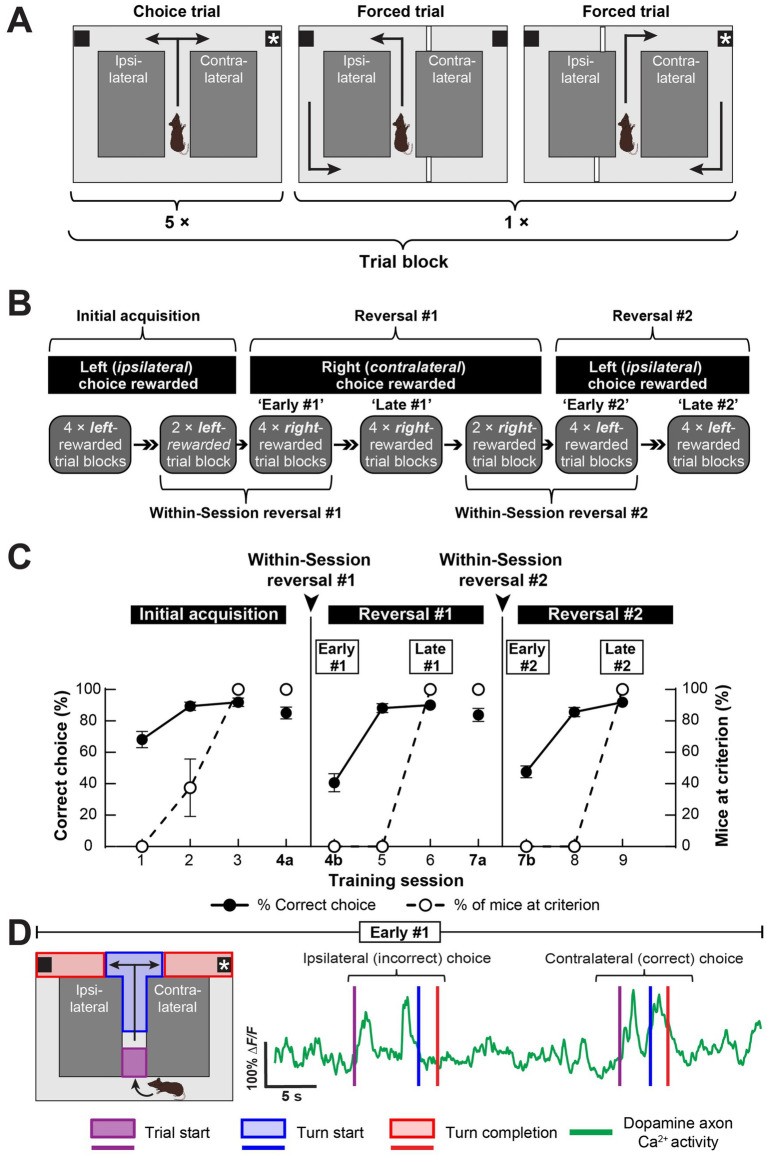
Imaging dopamine axon Ca^2+^ activity during reversal learning. **(A)** We trained mice in blocks of self-paced trials consisting of 5 choice and 2 forced trials in an automated T-maze. Note that we imaged dopamine neuron axon Ca^2+^ activity in the left hemisphere of all mice. **(B)** During initial learning, we trained mice to turn left in the maze to obtain a food reward. Once mice reached the performance criterion, the next day they underwent a “within-session” reversal consisting of 2 trial blocks in which left turns were rewarded, followed by 4 right-rewarded trial blocks. We refer to the first 4 right-rewarded trial blocks of the first within-session reversal as “Early #1”. We continued training the mice with 4, right-rewarded trial blocks per day until they reached criterion again. We refer to the last of these 4 trial blocks as “Late #1”. The next day, the mice underwent a second within-session reversal consisting of 2 trial blocks in which right turns were rewarded, followed by 4 left-rewarded trial blocks. We refer to the first 4 left-rewarded trial blocks of this second, within-session reversal as “Early #2.” We continued training the mice with 4 left-rewarded trial blocks per day until they reached criterion again. We refer to the last of these 4 left-rewarded trial blocks as “Late #2”. **(C)** Mice quickly learned the reversal learning task. All mice reached criterion within 2 sessions of the within-session reversal (data are mean ± s.e.m.; *N* = 9 mice). **(D)** Schematic of task epochs (*left*) and a representative mouse’s DMS dopamine axon Ca^2+^ activity trace showing dynamics with respect to the task epochs for two trials in the Early #1 reversal session (*right*). Refer also to Supplementary Videos S1, S2.

To determine the dopamine axon Ca^2+^ dynamics associated with reversal learning, we recorded the dynamics over multiple days in a serial reversal learning task in an automated T-maze. We trained the mice in blocks of 7 trials, each consisting of 5 choice and 2 forced trials in each direction (Figure 2A). We initially trained mice in sessions of four 7-trial blocks to turn into the left arm to obtain a food reward (Figure 2B). During this training, we did not reward turns to the right maze arm on either choice or forced trials. Notably, we implanted and imaged dopamine axon terminals in the left hemisphere of all mice, so the mice initially learned to turn ipsilaterally to the implanted hemisphere. We trained the mice until they turned left on ≥70% of the 20 choice trials in a session.

Following the initial acquisition, mice received the first “within-session” reversal. During this session, mice received two 7-trial blocks, in which we rewarded left turns (the initially rewarded arm), followed by four 7-trial blocks, in which we rewarded right instead of left turns (Figure 2B). After the within-session reversal session, we proceeded to train the mice in daily sessions of four 7-trial blocks in which we rewarded turns into the right but not the left maze arm. Once mice turned right on ≥70% of their choice trials, they underwent a second within-session reversal consisting of two 7-trial blocks, in which we rewarded right turns, followed by four 7-trial blocks, in which we rewarded left instead of right turns. After this second, within-session reversal, we continued training the mice in daily sessions of four 7-trial blocks with rewarded left but not right turns until they made ≥70% correct choices (Figure 2B).

The mice quickly learned the initial acquisition and both reversals, with all imaged mice reaching criterion in three sessions of each training phase (Figure 2C). During the within-session reversals, mice retained the previous reward contingency, and then, their % correct choice dropped to <50% on average upon reversal (Figure 2C).

To examine task-related dopamine axon Ca^2+^ activity, we used the maze’s infrared sensors to time-lock our miniature microscope recordings to the start of each trial, each forced or choice turn, and to the completion of each turn, which coincided with reward delivery or omission (Figure 2D). We observed robust dopamine axon activation when the pneumatic door opened at the start of each trial (Figure 2D). However, dopamine axon activation during trial initiation did not vary for ipsilateral versus contralateral choices either early or late in reversal learning (Supplementary Figure S1). By contrast, dopamine axon activation at the time of choice turn start differed for ipsilateral and contralateral turns on choice trials (Figure 2D). Furthermore, differences in dopamine axon activation at the time of completion (i.e., reward delivery or omission) also suggested choice direction-specific dopamine axon Ca^2+^ dynamics (Figure 2D). Therefore, we focused on these task epochs over the course of reversal learning in our analyses: dopamine axon Ca^2+^ dynamics during (1) trial start, (2) turn start, and (3) turn completion and their differences between ipsilateral and contralateral choices (Figure 2D; Supplementary Videos S1, S2).

### Choice-related dopamine axon dynamics across reversal learning

**Figure 3:**
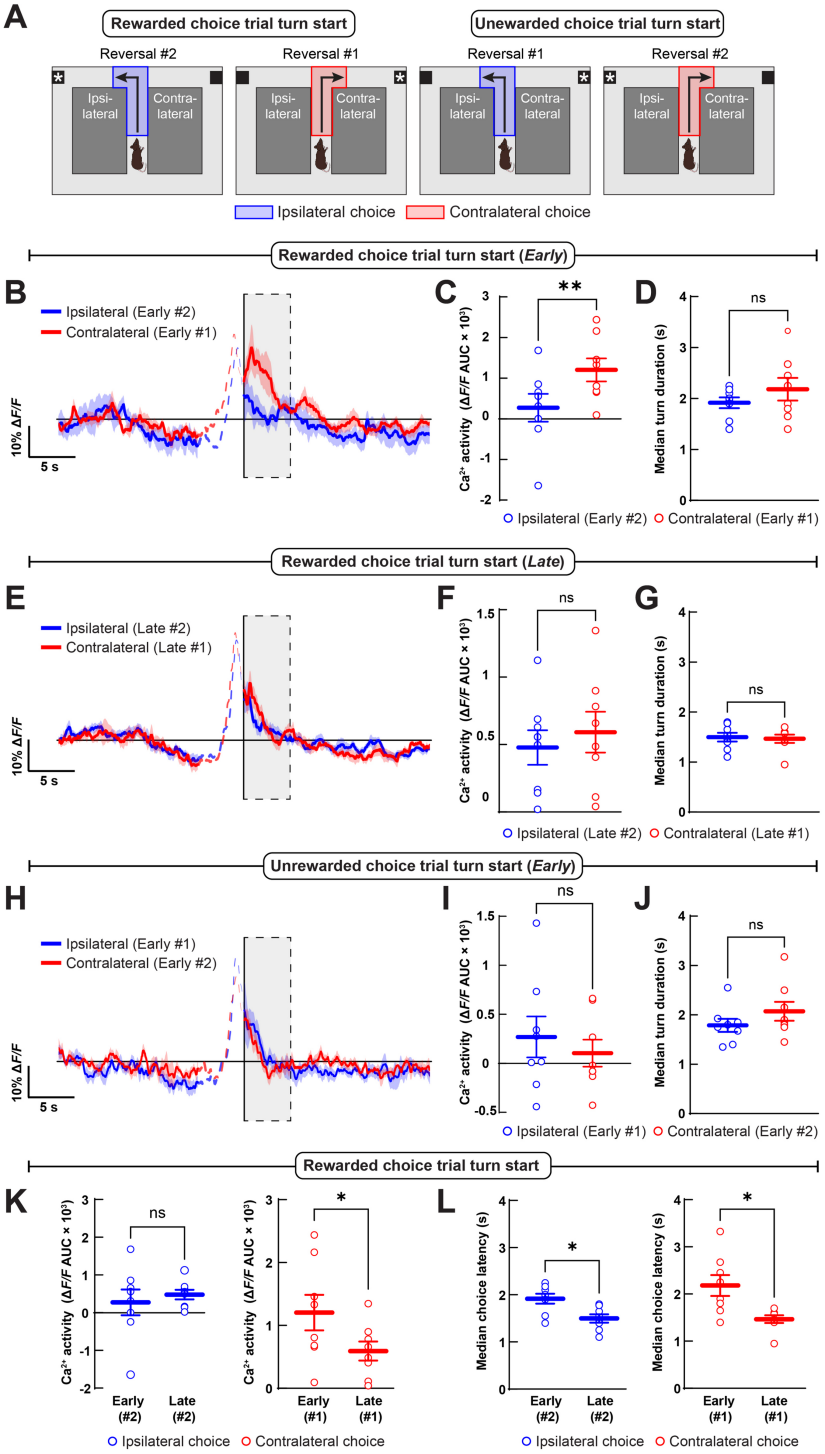
Dopamine may bias contralateral choice early in reversal learning. **(A)** We analyzed ipsilateral (*blue*) and contralateral (*red*) turn start epochs for rewarded and unrewarded choice trials, early and late in reversal learning. **(B,C)** Choice-related dopamine neuron axon Ca^2+^ activity in the DMS was significantly greater when the new correct choice direction was contralateral to the implanted hemisphere early in reversal learning, **(B)** during the 5 s after turn start in the 4-trial block of within-session reversals 1 and 2, as measured by the area under-the-curve (AUC), **(C)** of the Δ*F/F* trace. **(D)** There was no difference between the averages of each mouse’s median turn duration for correct ipsilateral and contralateral choices during this early block of reversal learning. **(E–G)** We found no difference in contralateral versus ipsilateral choice-related dopamine axon Ca^2+^ activity, **(E,F)** or turn duration, **(G)** later in reversal after the new contingency had been learned. **(H–J)** There were also no differences between ipsilateral and contralateral choice-related dopamine axon Ca^2+^ activity, **(H,I)** or turn duration, **(J)** when mice chose the previously rewarded (and now unrewarded) in the first sessions of each reversal. **(K)** There was a decrease in contralateral but no change in ipsilateral turn-related dopamine axon Ca^2+^ activity during rewarded choice trials over the course of reversal learning. **(L)** The duration of both ipsilateral and contralateral turns on rewarded choice trials decreased over the course of reversal learning. Data in **B–L** are mean ± s.e.m. of *N* = 8 mice. *p < 0.05 and **p < 0.01; Wilcoxon signed-rank test.

To determine how DMS dopamine may modulate action selection, we quantified dopamine axon activation during turn initiation on free choice trials as a function of turn direction and the phase of reversal learning (Figure 3A). First, we compared the dopamine axon activation during the initiation of contralateral (right) and ipsilateral (left) turns, immediately after each turn direction became rewarded during the first and second, within-session reversals (“Early 1” contralateral and “Early 2” ipsilateral choice trial turns; see Figures 2B,C). During these turns into the newly rewarded maze arm, dopamine axon activation was greater for contralateral than for ipsilateral turns (Figures 3B,C). Differences in movement vigor during turn initiation did not explain the greater dopamine axon activation during contralateral turns, as turns into either newly rewarded maze arm were of equal duration (Figure 3D). By contrast, dopamine axon activation during rewarded contralateral and ipsilateral choices was indistinguishable later in reversal, when mice preferred those turn directions (Figures 3E,F). There were also no temporal differences between these well-learned, correct choices (Figure 3G). There were no differences between dopamine axon activation or turn duration for ipsilateral and contralateral choices when each became newly unrewarded in early reversal (Figures 3H–J). The loss of a difference between ipsilateral and contralateral choice-related dopamine axon activation between early and late phases of reversal was driven by a learning-dependent decrease in contralateral, rather than a change to ipsilateral choice-related activation (Figure 3K). This change was also not explained by changes in movement vigor, as both choice directions became faster later in reversal learning (Figure 3L). We conclude that dopamine axon activation at the time of turn initiation only differed for contralateral and ipsilateral turns at the time that these turns became newly rewarded. Specifically, dopamine axon activation was greater during the initiation of contralateral turns, early in reversal to the contralateral maze arm, and then subsided as mice learned the new contingency.

### Choice outcome-related dopamine axon dynamics across reversal learning

**Figure 4:**
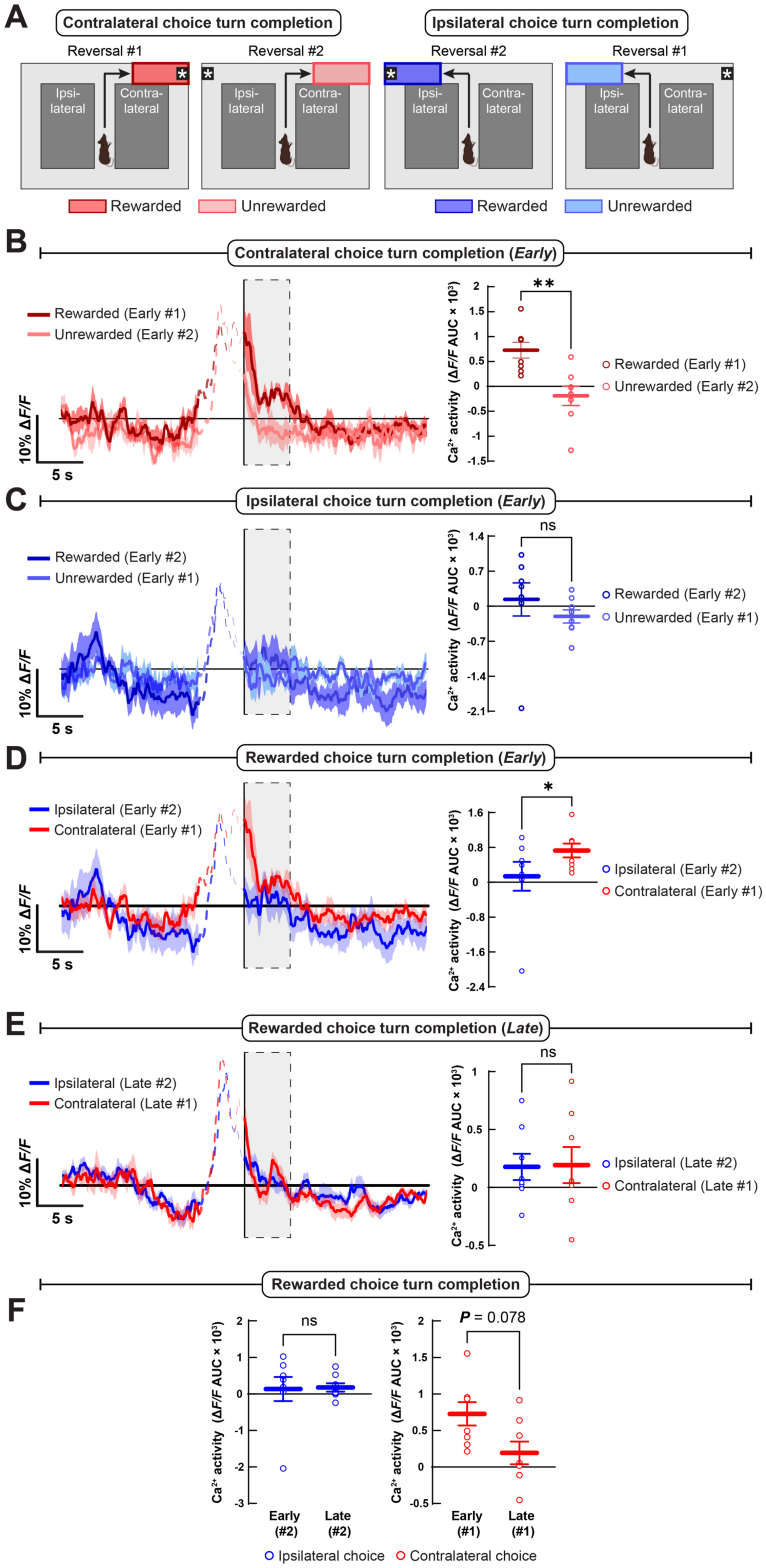
Reward delivery induces greater dopamine axon Ca^2+^ activation than reward omission following contralateral but not ipsilateral choices. **(A)** We time-locked dopamine axon Ca^2+^ dynamics to turn completion (i.e., reward delivery or omission) following choice trials during reversal learning. **(B)** After contralateral choices, unexpected rewards (*Early #1*) evoked greater dopamine axon Ca^2+^ activity than unexpected reward omission (*Early #2*) early in reversal learning. **(C)** Dopamine axon Ca^2+^ activity was indistinguishable for unexpected reward delivery and omission following choices that were ipsilateral to the imaged hemisphere early in reversal learning. **(D,E)** Reward delivery-evoked dopamine axon Ca^2+^ activity was greater following rewarded contralateral than ipsilateral choices early, **(D)** but not late, **(E)** in reversal learning. **(F)** Reward delivery-evoked dopamine axon Ca^2+^ activity did not vary following ipsilateral choices and non-significantly decreased following contralateral choices across reversal learning. All statistical comparisons are the Wilcoxon signed-rank test (*p < 0.05 and **p < 0.001). Data in **B–F** are mean ± s.e.m. of *N* = 8 mice.

Next, we asked whether DMS dopamine transmission encodes choice outcome (i.e., reward delivery or omission) and whether this differs for contralateral or ipsilateral choices (Figure 4A). Early in reversal learning, dopamine axon activation was greater at the time of unexpected reward delivery than unexpected reward omission following contralateral, but not ipsilateral choices (Figures 4B,C). This difference between dopamine axon activation at the end of correct contralateral and ipsilateral choices was specific to the early phases of reversal in each direction and did not occur once the mice reached criterion (Figures 4D,E). This decrease in lateralized, outcome-related dopamine axon Ca^2+^ activity appeared to result from a reward-prediction error-like decrease in reward delivery evoked activity following contralateral, but not ipsilateral choices over reversal learning, though the decrease did not reach significance (Figure 4F; though see also Supplementary Figure S4F).

In summary, DMS dopamine axon activation was greater during rewards than reward omissions, but only when the unexpected rewards were unexpected following a contralateral choice. This difference was driven by a greater increase in activation at the time of unexpected reward delivery, rather than by a reduction in activation during reward omission, suggesting that dopamine axons encode positive, but not negative, prediction errors in an action-selective manner.

### Choice- and outcome-related dopamine axon dynamics differ between free and forced choice trials

To determine whether these direction-selective, DMS dopamine axon activation dynamics during turn and outcome depend on choice volition, we analyzed the same epochs during the interspersed forced trials, early and late in reversal learning (Supplementary Figures S3A, S4A). The greater increase in DMS dopamine axon activation during contralateral choice turns was absent both early and late in reversal learning during rewarded forced choices (Supplementary Figures S3B–G). There wera also no directional differences between dopamine axon activation the initial unrewarded forced choices early in reversal learning (Supplementary Figures S3H–J). The durations of ipsilateral and contralateral choices also did not differ for the different forced trial types. Unlike for choice trials (Figure 3K), contralateral forced choice-related dopamine axon activation did not decrease over the course of reversal learning (Supplementary Figure S3K), even though the duration of these correct forced choices decreased similarly to correct free choices as mice learned the new contingency (Figure 3L; Supplementary Figure S3L).

In contrast to choice direction-related dopamine axon activation, choice direction outcome-related dopamine axon activation was more similar between choice and forced trials over the course of reversal learning. However, following forced trial turns, dopamine axon activation was greater for unexpected reward delivery than omission following both ipsilateral and contralateral forced choices (Supplementary Figures S4A–C). The same as for choice trials, dopamine axon activation following reward delivery upon forced choice completion was greater for contralateral than ipsilateral choices, but only early in reversal learning (Supplementary Figures S4D,E). Reward delivery-evoked dopamine axon Ca^2+^ activation decreased over learning following contralateral, but not ipsilateral forced choices, and reward omission-evoked activation did not change following either choice direction over the course of reversal learning (Supplementary Figures S4F,G).

These results suggest that dopamine axon Ca^2+^ activity exhibits reward-prediction error-like dynamics following contralateral, but not ipsilateral, forced choices. Moreover, the directional selectivity of choice action-related dopamine axon dynamics depends more upon volition (i.e., free choice versus forced choice) than action outcome-related dopamine axon dynamics.

### Optogenetic inactivation of SNc dopamine neurons during reversal learning

**Figure 5:**
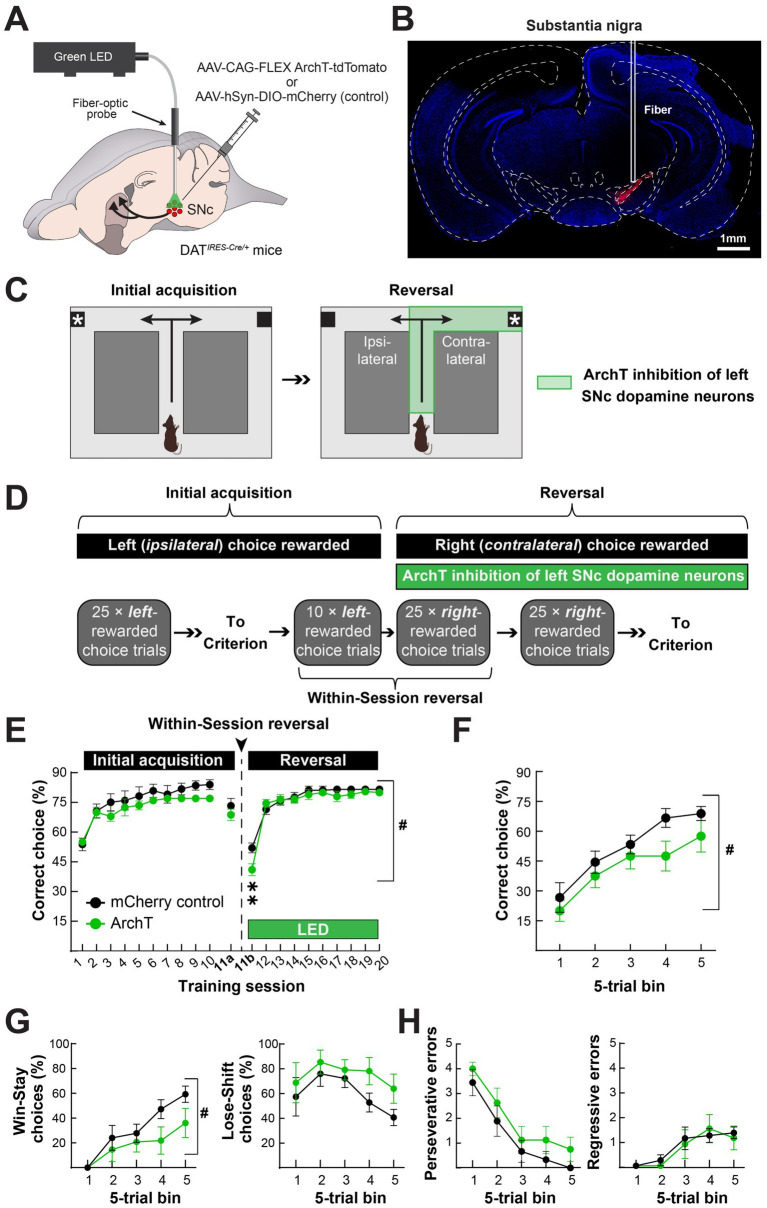
Optogenetic inhibition of the nigrostriatal dopamine pathway attenuates contraversive reversal at the time of contingency switch. **(A)** We unilaterally expressed the inhibitory, light-gated proton pump ArchT in SNc dopamine neurons of DAT-Cre mice and implanted a fiber-optic for optogenetic inhibition. **(B)** Histological verification of unilateral ArchT expression in the SNc in a representative mouse. **(C)** We initially trained mice to turn left (*ipsilaterally*) to obtain a food reward. Once the mice reached stable performance, they underwent reversal in conjunction with optogenetic inhibition throughout the turn initiation and completion phases (i.e., encompassing the periods in Figures 3B, 4B). **(D)** For initial training, mice received 25 choice trials per session across 10 days in which the left (*ipsilateral*) choice was rewarded in the absence of any light stimulation. On the day of the reversal, mice received a within-session reversal consisting of 10 left-rewarded choice trials with no light stimulation, followed by 25 right-rewarded choice trials in conjunction with light stimulation. We subsequently trained mice with 25 right-rewarded choice trials and light stimulation until they reached criterion. **(E)** There were no differences in performance between control (SNc-mCherry) and experimental (SNc-ArchT-tdTomato) mice during initial acquisition or the first 10 trials of the within-session reversal, as measured by % correct choices and mice at criterion (*left*). Upon light stimulation, experimental mice performed worse than control mice, but only during the first 25 trials upon contingency reversal (^#^*p* = 0.012; two-way ANOVA Group × Time effect; **p < 0.005; Holm-Sidak multiple comparisons). **(F–H)** During the first 25 right (*contralateral*) choice-rewarded trials with light stimulation in the within-session reversal, ArchT inhibition of SNc dopamine neurons reduced the number of correct choices, **(F)**, and the number of “Win-Stay” choices, **(G)** (*left*), but had no effect on the numbers of “Lose-Shift” choices, **(G)** (*right*), or perseverative, **(H)** (*left*), or regressive errors, **(H)** (*right*) (^#^*p* < 0.05; two-way ANOVA Group effect). Data in **E–H** are mean ± s.e.m. of *N* = 9 control and *N* = 8 experimental mice.

To determine how the augmented nigrostriatal dopamine pathway activity during turn initiation and completion influences reversal learning, we unilaterally expressed the inhibitory opsin ArchT (Okazaki and Takagi, 2013) in the left SNc dopamine neurons of DAT-Cre mice and implanted a fiber-optic cannula just above the injection site (Figure 5A). This approach expressed ArchT-tdTomato in the DMS and in SNc dopamine neurons at the site of the fiber optic implant Figure 5B). We generated control mice the same way but used an mCherry-expressing virus.

We trained these mice to initially turn left (ipsilateral to the implanted hemisphere) in the T-maze to obtain reward and then used optogenetic inhibition to determine how inhibiting the nigrostriatal dopamine pathway activation we observed during and after the turn affected their ability to learn to turn right upon reversal Figure 5C). Specifically, we trained the left hemisphere-implanted mice to turn left to obtain a food reward in daily sessions of 25 choice trials. Once the mice made ≥ 70% correct choices, they underwent a within-session reversal the subsequent day Figure 5D). For the first 10 trials of the within-session reversal, we continued rewarding only left turns in the maze without any defined in methods stimulation. For the last 25 trials, we rewarded only right turns in the maze and inhibited SNc dopamine neurons for 5 s after turn initiation in either direction and then for another 5 s following turn completion, only after right choices (Figures 5C,D). We continued training the mice with the same optogenetic inhibition parameters in 25 right-rewarded choice trial sessions until they reached criterion (≥ 70% right choices) (Figure 5D).

Control (SNc-mCherry) and experimental (SNc-ArchT) mice equivalently learned the initial acquisition of turning left in the maze to obtain the food reward Figure 5E). The slower rate of learning in these experiments, compared to the *in vivo* imaging mice, may be attributed to the lack of the interspersed forced trials in the training procedure (comparing Figures 5E, 2C). After reaching criterion, both groups retained their criterion level of % left-turn choices in the first phase of the within-session reversal (10-trial block of left choice-rewarded trials in session 11a). However, when we reversed the reward contingency to the right maze arm, optical stimulation decreased right-arm choices in experimental but not control mice (Figure 5E). This attenuation in reversal to turning in the direction contralateral to the inhibited SNc was specific to the first, within-session reversal (Figures 5E,F). After the within-session reversal, reversal learning was indistinguishable between the groups, despite optogenetic inhibition (Figure 5E). The early deficit in reversal learning following optogenetic inhibition of SNc dopamine neurons was driven by a deficit in “win-stay,” rather than “lose-shift” decisions, suggesting that experimental mice had difficulty maintaining their contralateral choice strategy after the choice became newly rewarded. Despite this deficit, experimental and control mice similarly reduced their “perseverative” errors (≥ 3 left/ipsilateral choices in a 5-trial block) and had a similar number of “regressive” errors (left/ipsilateral choices after perseverative errors ceased) (Figures 5G,H). In conclusion, optogenetic inhibition of SNc dopamine neurons during turn initiation and outcome attenuated reversal learning specifically during the phase of reversal learning when nigrostriatal dopamine pathway activation was increased in *in vivo* recordings.

## Discussion

In this study, we used a miniature microscope to record dopamine axon Ca^2+^ activity in the DMS of awake, behaving mice. Dopamine neuron axon activation increased during motion onset and decreased during motion offset during exploration of an open field arena. Next, we recorded Ca^2+^ activity in dopamine neuron axons during reversal learning in a T-maze. In the first session following rule reversals, we observed lateralized differences in dopamine axon activation at the time of choice initiation and completion (i.e., reward delivery or omission). Specifically, dopamine axon activation was greater during and after contralateral than ipsilateral turns, but only during the first session in which contralateral turns became newly rewarded.

### Lateralized, choice-related dopamine responses

The activation of midbrain dopamine neurons and dopamine axons in the dorsal striatum is unequivocally linked to movement, but the precise contributions of dopamine signaling to movement remain under active investigation (Howe and Dombeck, 2016; da Silva et al., 2018; Coddington and Dudman, 2019). A functional lateralization of dopamine’s contributions to movement has long been recognized, as evidenced by the classical unilateral dopamine depletion model for Parkinson’s disease. In this animal model, unilateral dopamine depletion induces an ipsilateral rotational bias that becomes contralateral following treatment with dopamine receptor agonists or the dopamine biosynthetic precursor L-DOPA (Schwarting and Huston, 1996; Parker et al., 2018; Yu et al., 2022). Consistent with this finding, the unilateral optogenetic inhibition of SNc dopamine neurons induces ipsilateral bias, and unilateral optogenetic activation progressively entrains contralateral rotations in response to a cue paired with activation (Mahn et al., 2021; Engel et al., 2024).

*In vivo* measurements have shown that dopamine axon activation and dopamine release in the DMS is greater for contralateral than for ipsilateral movements (Parker et al., 2016; Hart et al., 2024; Mendonça et al., 2024). Moreover, midbrain dopamine neuron activation scales with the duration of contralateral but not ipsilateral motor sequences, perhaps contributing to the contralateral movement’s vigor (Mendonça et al., 2024).

Our results contribute to the emergent understanding of dopamine’s role in action selection. In particular, we found that choice-related dopamine axon activation in DMS was only greater for the contralateral choices at the first moment of reversal learning when the contralateral choice becomes rewarded (Figure 3B). Later, after the mice had learned the new action-outcome contingency, contralateral choice dopamine axon activation subsided and became indistinguishable in magnitude from ipsilateral choices (Figure 3C). Our results suggest that lateralized dopamine transmission may bias action toward contralateral choices, specifically when the ipsilateral choice-outcome contingency becomes conflicted. Our optogenetic inhibition experiments support this interpretation (Figures 5E,F).

Our findings are consistent with another study that used a lateralized, lever-pressing task to assess the reversal of probabilistic reward delivery (Parker et al., 2016). Their within-session, serial reversal design caused an ever-present conflict in choice-outcome contingency. In our data, contralaterally biased dopamine axon activation was greatest in the first session reversal to the contralateral maze arm, a period of high choice-outcome contingency conflict. In addition, consistent with our data, Parker *et al.* found that dopamine release was negatively modulated by reward receipt on the previous trial. Another study reported that a contralateral bias in choice-related DMS dopamine release emerges later in choice-outcome contingency learning (Hart et al., 2024). In their study, the contralateral bias arose as the reinforcement schedule progressed to one with greater choice-outcome contingency (i.e., a 30-s random-interval reinforcement schedule). The authors report that choice-related dopamine release becomes biased for contralateral choices when reward expectation is greatest. However, in their modeling, reward expectation rises just after reward receipt, which corresponds to the least likely period of reward receipt in a random-interval reinforcement schedule. It is also notable that their task consisted of only forced choices (i.e., the left and right levers were presented in isolation across trial blocks). Therefore, the subjects were never faced with competing choices, as in our study and the study of Parker *et al.* Taking these experimental design differences into account, our results are consistent with these earlier studies and support dopamine’s role in biasing choice direction under conditions with elevated action-outcome contingency conflict.

### Lateralized, outcome-related dopamine responses

Following the outcome of a given action, dopamine neurons canonically encode reward-prediction errors (Schultz et al., 1997). The phasic activation of dopamine neurons in response to better-than-expected outcomes and phasic suppression following worse-than-expected outcomes are believed to drive learning about the outcome’s antecedent actions or cues (Pan et al., 2005). In rodent studies, reward-prediction errors prevail in the ventral striatum, yet dopamine also encodes outcome in the DMS and may facilitate the structure’s purported function in action-outcome learning. Previous studies recording dopamine axon activation or release in the DMS suggest that there are reward-evoked dopamine responses in the DMS, though smaller in magnitude than in the ventral striatum (Tsutsui-Kimura et al., 2020). A majority of studies support the presence of reward-related dopamine prediction errors in the DMS (Parker et al., 2016; Tsutsui-Kimura et al., 2020; Hollon et al., 2021; Mohebi et al., 2024). Reward prediction error-like outcome coding was reported in another study, specifically following contralateral choices (i.e., a difference between reward receipt and omission), though this did not scale with reward expectation in their model (Hart et al., 2024).

One explanation for our findings is that dopamine axon activation and release in the DMS becomes reduced for outcomes when the action-outcome contingency is consistent (Tsutsui-Kimura et al., 2020; Hollon et al., 2021). In concordance, reward-evoked dopamine release was greatest in our study in the first reversal session, right when the action-outcome contingency changed. Interestingly, we only observed this effect when the new reward contingency was for contralateral action (Figures 4B–D). The lateralization of this effect—increased dopamine following newly rewarded actions contralateral to the recorded hemisphere—adds a new dimension (i.e., directionality) with which DMS dopamine may promote new action-outcome contingencies. Specifically, DMS dopamine did appear to encode a reward-prediction error in our study, but did so in a lateralized manner, preferentially following contralateral actions that resulted in unexpected outcomes. This lateral specificity may be important for linking specific outcomes to actions, which may explain why unilaterally inhibiting the nigrostriatal dopamine pathway reduced win-stay responses in the first session of reversal to the contralateral maze arm in our study (Figures 5E–G).

### Limitations and additional considerations

While our experimental design allowed for an explicit evaluation of dopamine axon dynamics during reversal learning, there were several limitations to the study. One possible caveat is that we did not vary the directional ordering of the reversals (Figures 2B,C). All mice were initially trained to turn ipsilaterally in the maze; the first reversal was always to the contralateral maze arm, and the second back to the ipsilateral maze arm. In this context, the rigid ordering of reversal directions may have contributed to the lateralized differences in dopamine axon activation we observed, rather than the directions themselves. For instance, the contralateral bias in dopamine axon activation we observed early in the first reversal could have been due to the fact that it was the first reversal, rather than the fact that the reversal was to the side contralateral to the recorded hemisphere. Related to this limitation, we were unable to record dopamine axon activity bilaterally in the same mice due to the size of the miniature microscope. Future studies using more compact neural imaging approaches like fiber photometry could overcome this limitation.

In this study, we treated the entire field-of-view as a singular region-of-interest, but future analyses of the spatiotemporal dynamics across the imaging field could provide additional insights into the role of DMS dopamine signaling in reversal learning. For example, one could further analyze the wavelike dynamics of dopamine axon activation to determine whether these dynamics correlate with changes in task strategy (Hamid et al., 2021). Similarly, although we could not resolve individual dopamine axons using this approach, higher resolution imaging (i.e., imaging without a GRIN lens) could potentially reveal any underlying heterogeneity in dopamine axon activation that may also map onto task-relevant behavior (Howe and Dombeck, 2016).

In our optogenetics experiment, we transduced and implanted our fiber optic over the SNc, without specificity for projections to the DMS. Future experiments manipulating projection-defined SNc dopamine neurons are necessary to causally link dopamine transmission in DMS (or other dorsal striatal regions) to reversal learning. Furthermore, we unilaterally inhibited SNc dopamine neurons throughout the choice initiation and completion phases (i.e., both action selection and outcome). Future experiments separating the two could be useful for determining which aspect of contralateral dopamine transmission may be more important for reversal learning. We also did not examine whether there were any effects on reversal to the maze arm ipsilateral to the unilateral SNc inhibition, nor did we test the effects of unilateral SNc dopamine neuron activation, which might be predicted to promote reversal to the maze arm contralateral to the implant. Finally, although it has been shown by others to suppress dopamine release *in vivo* (Danjo et al., 2014; Chen et al., 2025), we did not validate that ArchT-mediated inhibition of SNc dopamine neurons diminished dopamine axon activation or release in the DMS in our mice. We only verified the targeting of virus expression and fiber optic placement in the SNc (Figure 5).

## Data Availability

The raw data supporting the conclusions of this article will be made available by the authors, without undue reservation.

## Footnotes

1
https://bahanonu.github.io/ciatah/
