# Supplementary material for: Lateralized nigrostriatal dopamine pathway activation promotes early reversal learning
Source: Front Behav Neurosci. 2025 Dec 10;19:1703094. doi: 10.3389/fnbeh.2025.1703094 (PMC12727883; doi:10.3389/fnbeh.2025.1703094)
Supplement: Supplementary file 3 [file Data_Sheet_1.docx]

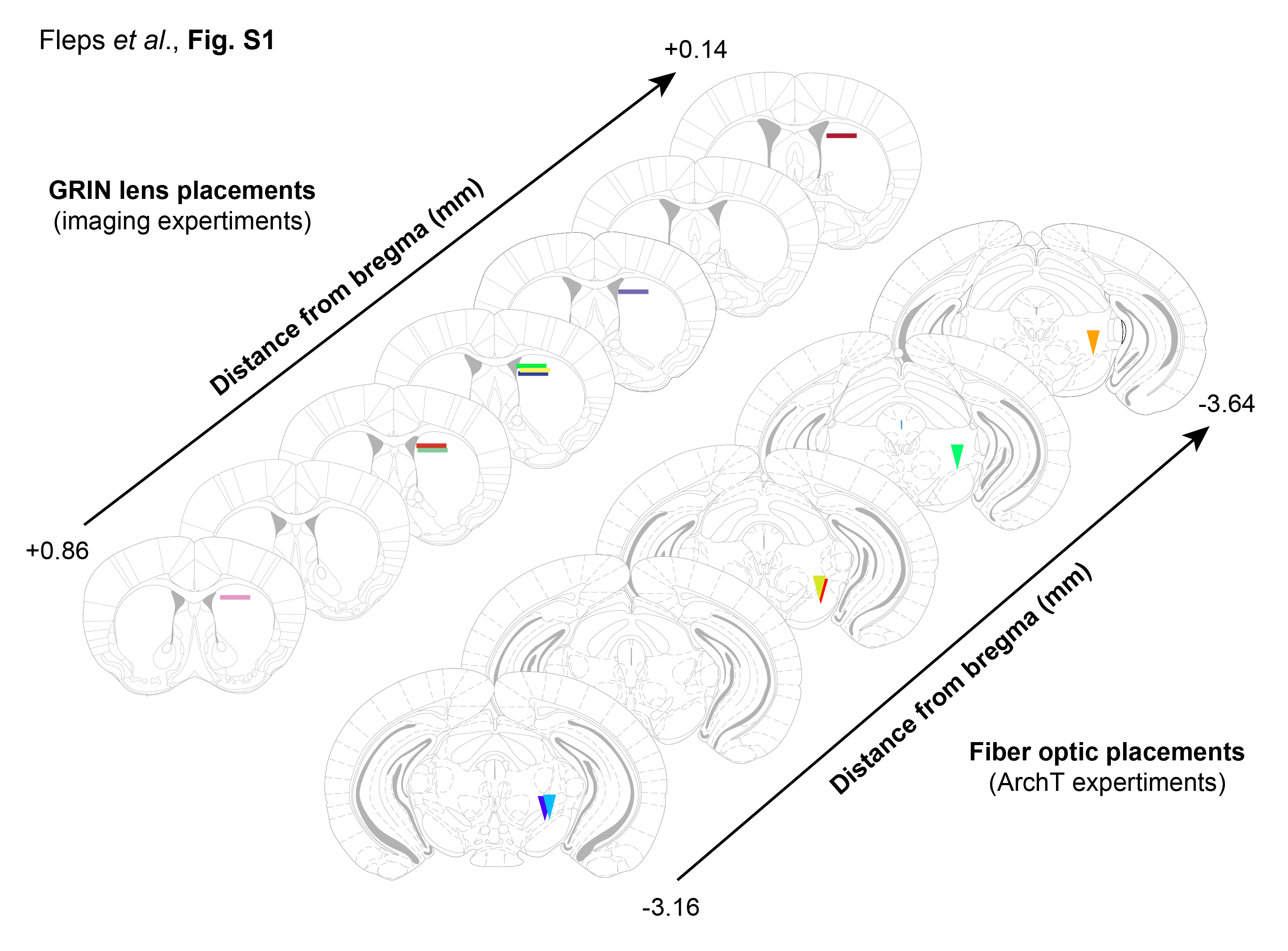


**Figure S1. GRIN lens and fiber-optic implant sites.** Histologically verified GRIN lens placements for *in vivo* imaging (*top*) and optogenetic inhibition experiments (*bottom*). Different color markers denote individual mice overlaid on mouse brain atlas sections.


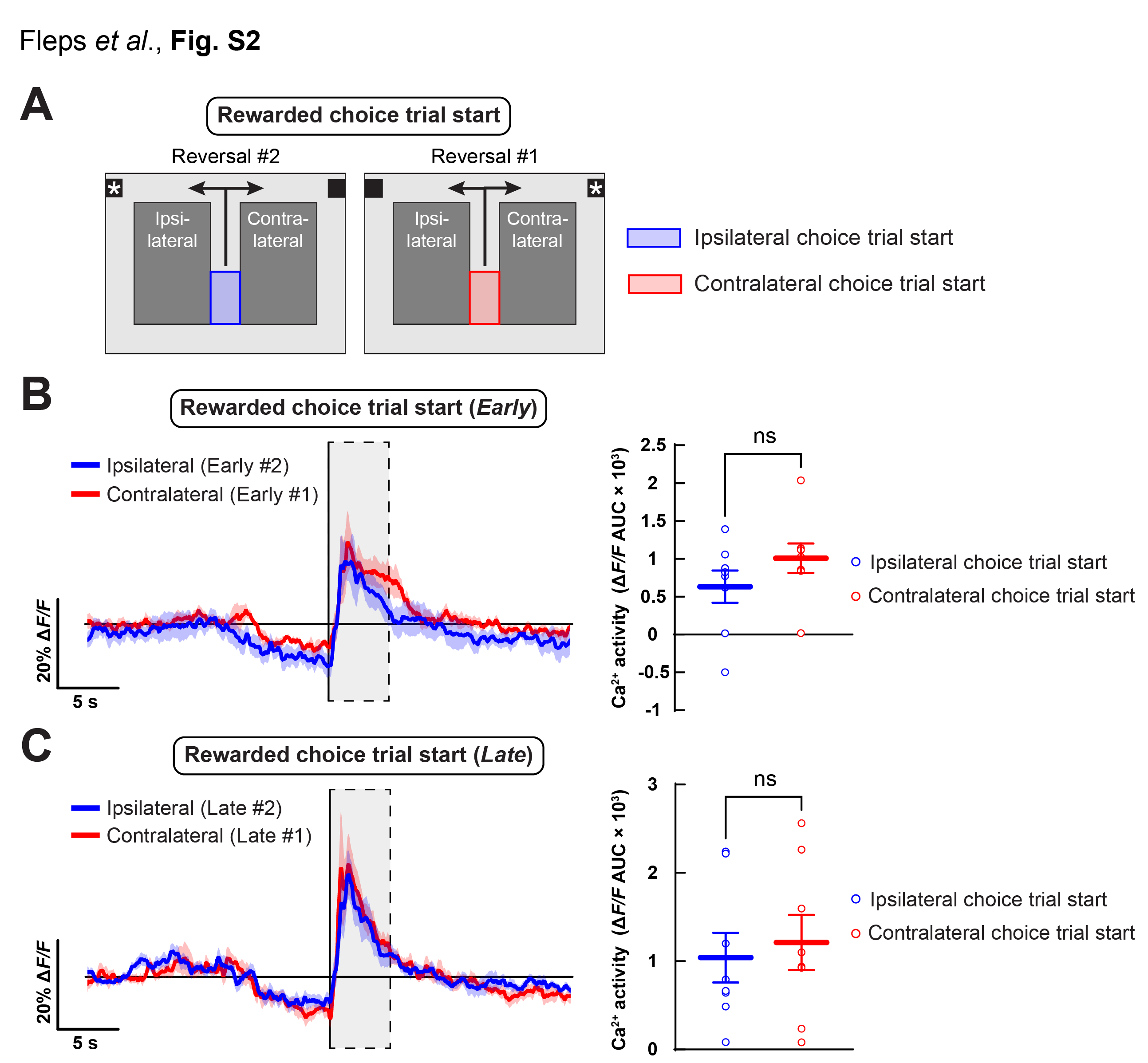


**Figure S2. Trial initiation-evoked dopamine axon Ca^2+^ activity does not differ for different task contingencies or stages of reversal learning. A)** Schematic depiction of the task epoch to which we time-locked dopamine axon Ca^2+^ activity during Reversals #1 and #2. Each trial started with the pneumatic lowering of the maze start-arm door**. B, C**) In both the early, **B**, and late, **C**, stages of reversal, trial start-evoked dopamine axon Ca^2+^ activity in the DMS that did not differ for the ipsilateral versus contralateral task contingencies. Data in **B**, **C** are mean ± s.e.m. of *N* = 8 mice.


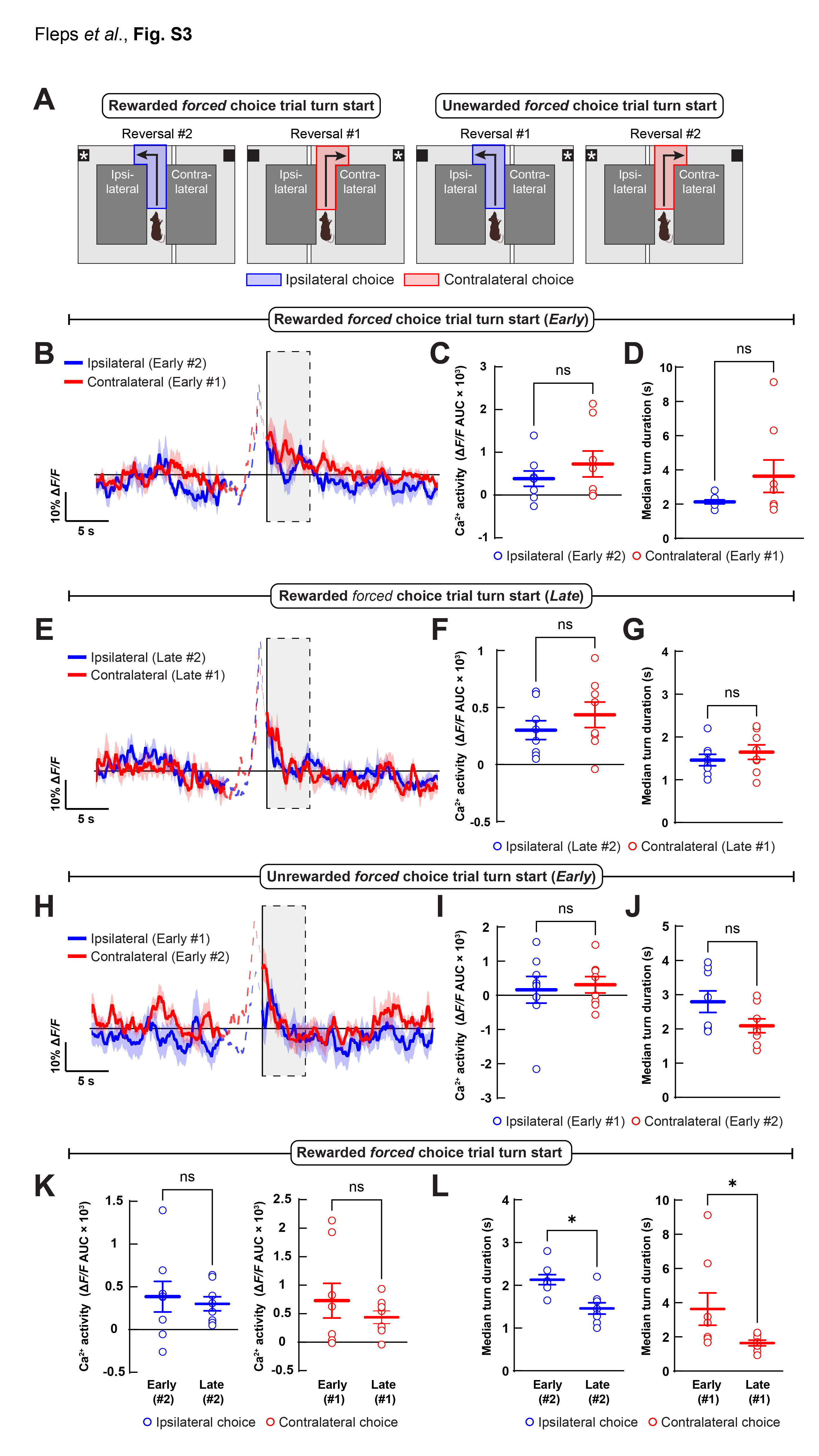


**Figure S3. Dopamine axon Ca^2+^ activation does not differ for ipsilateral and contralateral choices during forced trials. A)** We analyzed choice-related dopamine axon Ca^2+^ dynamics on forced trials, early and late in reversal learning. **B, C)** Forced choice-related dopamine neuron Ca^2+^ activation did not differ for rewarded forced choices in either direction during early reversal learning as measured by the AUC, **C**, of the Δ*F/F* trace, **B**, during the 5-seconds after turn start in the 4-trial block of within-session reversals 1 and 2. **D)** There was no difference between the median durations of each mouse’s ipsilateral and contralateral choices on rewarded forced trials during this early block of reversal learning. **E–G)** We found no difference in contralateral versus ipsilateral forced choice-related dopamine axon Ca^2+^ activity, **E**, **F**, or turn duration, **G**, later in reversal after the new contingency had been learned. **H–J)** There were also no differences between ipsilateral and contralateral forced choice-related dopamine axon Ca^2+^ activity, **H**, **I**, or turn duration, **J**, when mice chose the previously rewarded (and now un-rewarded) in the first sessions of each reversal. **K)** There was no change in either contralateral or ipsilateral turn-related dopamine axon Ca^2+^ activity on rewarded forced trials over the course of reversal learning. **L)** The duration of both ipsilateral and contralateral rewarded forced choice turns decreased over the course of reversal learning. Data in **B**–**L** are mean ± s.e.m of *N* = 8 mice. **P* < 0.05; Wilcoxon signed-rank test.


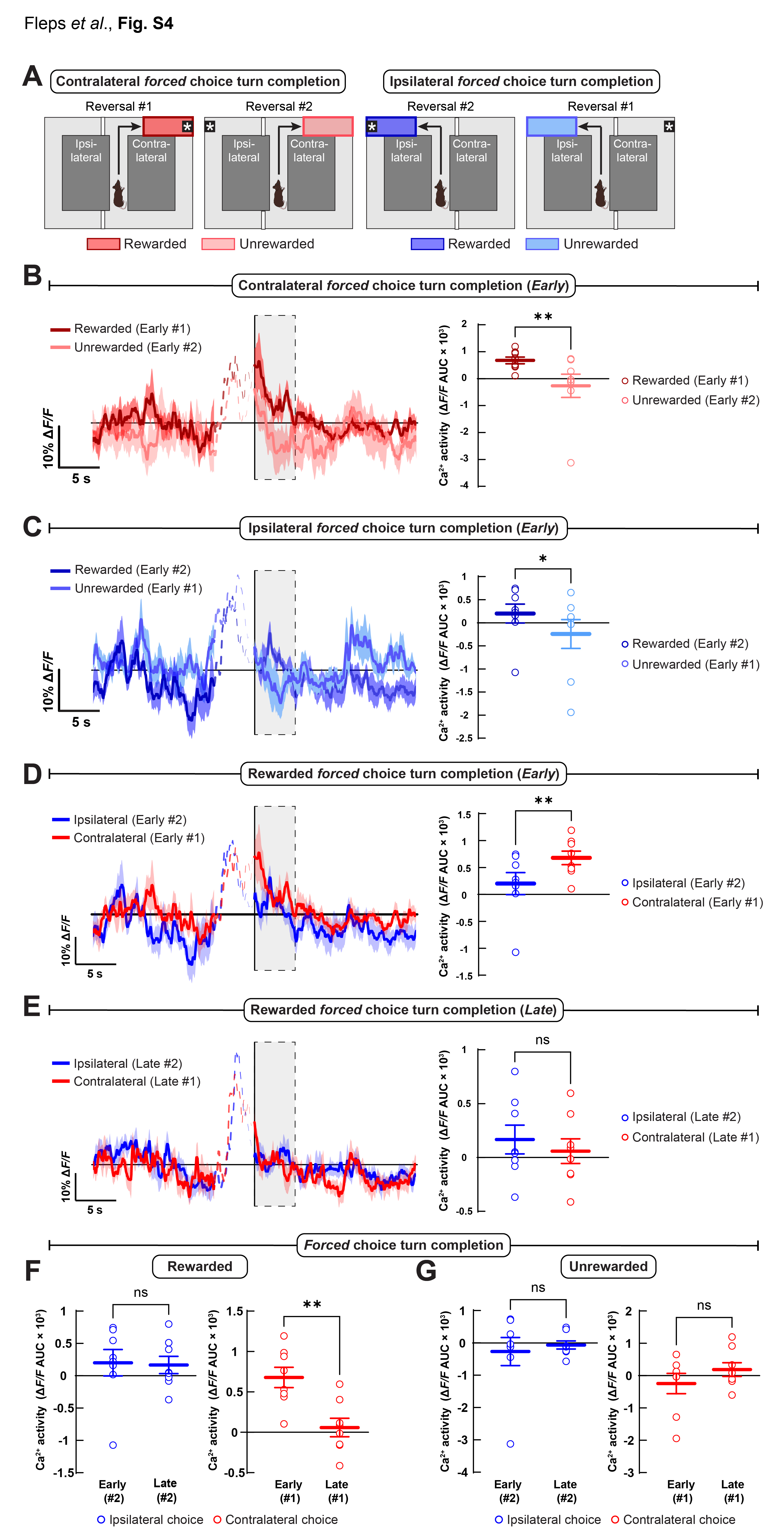


**Figure S4. Reward delivery induces greater dopamine axon Ca^2+^ activity than reward omission after contralateral choices, early in reversal learning on forced trials. A)** We time-locked dopamine axon Ca^2+^ dynamics to turn completion (*i.e*., reward delivery or omission) on forced trials during reversal learning. **B, C)** Unexpected rewards evoked greater dopamine axon Ca^2+^ activity than unexpected reward omission after both contralateral, **B**, and ipsilateral, **C**, forced choices early in reversal learning. **D, E**) Reward delivery-evoked dopamine axon Ca^2+^ activity was greater following rewarded contralateral than ipsilateral forced choices early, **D**, but not late, **E**, in reversal learning. **F)** Reward delivery-evoked dopamine axon Ca^2+^ activity decreased later in reversal for contralateral, but not ipsilateral forced choices. **G)** Reward omission-evoked dopamine axon Ca^2+^ activity following forced choices did not change over the course of reversal learning for either choice direction. All statistical comparisons are Wilcoxon signed-rank test (**P* < 0.05 and ***P* < 0.001). Data in **B–G** are mean ± s.e.m of *N* = 8 mice.

**Video 1. Dopamine axon Ca^2+^ dynamics during an incorrect, ipsilateral choice when contralateral choice becomes first rewarded (*Early #1*).** 20-Hz, Δ*F/F* movie of dopamine axon Ca^2+^ activity synchronized to behavior for a mouse making an incorrect choice to the previously rewarded ipsilateral arm during the second part of the first, within-session reversal (*Early #1*). Movie playback is 1.5× real time.

**Video 2. Dopamine axon Ca^2+^ dynamics during a correct, contralateral choice when contralateral choice becomes first rewarded (*Early #1*).** 20-Hz, Δ*F/F* movie of dopamine axon Ca^2+^ activity synchronized to behavior for a mouse making a correct choice to the newly rewarded contralateral arm during the second part of the first, within-session reversal (*Early #1*). Movie playback is 1.5× real time.
